# Fibroblasts: Heterogeneous Cells With Potential in Regenerative Therapy for Scarless Wound Healing

**DOI:** 10.3389/fcell.2021.713605

**Published:** 2021-07-20

**Authors:** Ming-Li Zou, Ying-Ying Teng, Jun-Jie Wu, Si-Yu Liu, Xiao-Yu Tang, Yuan Jia, Zhong-Hua Chen, Kai-Wen Zhang, Zi-Li Sun, Xia Li, Jun-Xing Ye, Rui-Sheng Xu, Feng-Lai Yuan

**Affiliations:** ^1^Wuxi Clinical Medicine School of Integrated Chinese and Western Medicine, Nanjing University of Chinese Medicine, Wuxi, China; ^2^Institute of Integrated Traditional Chinese and Western Medicine, The Affiliated Hospital of Jiangnan University, Wuxi, China; ^3^Institute of Integrated Traditional Chinese and Western Medicine, The Third Hospital Affiliated to Nantong University, Wuxi, China

**Keywords:** papillary fibroblasts, scarless wound healing, tissue repair, reticular fibroblast, dermal-subcutaneous junction fibroblasts

## Abstract

In recent years, research on wound healing has become increasingly in-depth, but therapeutic effects are still not satisfactory. Occasionally, pathological tissue repair occurs. Influencing factors have been proposed, but finding the turning point between normal and pathological tissue repair is difficult. Therefore, we focused our attention on the most basic level of tissue repair: fibroblasts. Fibroblasts were once considered terminally differentiated cells that represent a single cell type, and their heterogeneity was not studied until recently. We believe that subpopulations of fibroblasts play different roles in tissue repair, resulting in different repair results, such as the formation of normal scars in physiological tissue repair and fibrosis or ulcers in pathological tissue repair. It is also proposed that scarless healing can be achieved by regulating fibroblast subpopulations.

## Introduction

People suffer from a diverse range of injuries from the environmental, not only psychological and emotional injuries but also physical injuries. As the largest barrier of the human body, the skin protects us from external trauma, and in response, scars are formed. With economic development, the treatment of various types of hypertrophic scars has become a major burden on society (Trace et al., 2016). Scarless wound healing is often mentioned as an idea but is still an elusive goal. Generally, scar treatment methods include surgery, drugs, radiation and other physical therapies (Lee and Jang, 2018; Huang et al., 2019). However, most of their effects can at most achieve functional repair. Aesthetic results are basically ignored or are difficult to take into account. Thus, finding new treatments is imperative.

In theory, scar treatment should return the skin to the original starting point, perhaps starting from the smallest unit that constitutes the human body: cells. The skin is composed of the epidermis, the dermis and subcutaneous tissue (Gould, 2018). Scars only form when the dermis and subcutaneous tissues are injured (Jiang et al., 2020). Because the dermis is mainly composed of fibroblasts, our attention is focused on those cells.

For a long time, fibroblasts have been regarded as having a simple cell morphology. It has now been demonstrated that fibroblasts are actually a morphologically and functionally heterogeneous cell population (Mahmoudi et al., 2019). The establishment of fibroblast heterogeneity in a number of tissues using novel techniques represents a significant step forward in the fibroblast field. Studies have shown that fibroblasts in the dermis of the skin can be divided into papillary fibroblasts (Fps), reticular fibroblasts (Frs), and dermal-subcutaneous junction fibroblasts (F-DHJs) (Haydont et al., 2019, 2020). What function do they have in the process of tissue repair and scar formation? Is it possible that there is a group of cells that are specifically responsible for skin fibrosis? These questions need to be carefully addressed.

In this review, we summarize the heterogeneity of fibroblasts and the feasibility of clinical application. The results of our study may provide new insight into scarless wound healing.

## Structure of the Skin

The skin is the largest organ in our body and comprises three layers, namely, the epidermis, dermis and hypodermis, which have several primary functions, including protection, temperature regulation, secretion, excretion, sensation, and absorption (Dąbrowska et al., 2018). The skin helps maintain homeostasis, and healthy skin can reflect overall wellness (Matejuk, 2018).

The epidermis is the uppermost layer of the skin and acts as a physical barrier, preventing water loss from the body and stopping entry of foreign substances into the body (Baroni et al., 2012). It is made of four or five layers of epithelial cells, depending on its location on the body. The epidermis is mainly composed of three cell types, namely, keratinocytes (which account for the majority of epidermal cells), melanocytes and Langerhans cells (Kanitakis, 2002). In recent years, Merkel cells have been found in the basal layer of the epidermis, but their exact function is still unclear (Maksimovic et al., 2014).

The dermis is located between the epidermis and subcutaneous tissue and is composed of a variety of stromal cells. It is separated from the epidermis by the basal zone and has close contact with the other layers (Arda et al., 2014). The dermis is made of two layers of connective tissue that compose an interconnected mesh of elastin and collagenous fibers produced by fibroblasts (Le Digabel et al., 2018; Thulabandu et al., 2018). The dermis provides structure, strength and flexibility to the skin and houses other structures, such as blood capillaries, oil and sweat glands, nerve endings, and hair follicles. The resident cell type of the dermis is the dermal fibroblast, which produce extracellular matrix (ECM) and contribute to hair follicle initiation and cycling (Thulabandu et al., 2018).

The hypodermis, also called the subcutaneous layer or superficial fascia, is a layer directly below the dermis that serves to connect the skin to the underlying fibrous tissue of the bones and muscles (Khavkin and Ellis, 2011). The subcutis mainly consists of adipocytes, nerves and blood vessels (Prost-Squarcioni, 2006). Adipocytes are organized into lobules, which are separated by structures called septa. The septa contain nerves, larger blood vessels, fibrous tissue and fibroblasts. Thus, the hypodermis can function as a mode of fat storage and provide insulation and cushioning for the integument.

The above three layers constitute the largest protective barrier for the human body and provide protection from mechanical impacts, pressure, variations in temperature, microorganisms, radiation and chemicals.

## Correlation of the Dermis With Wound Healing and Scar Formation

The capacity of a wound to heal depends on many conditions (body location, sex, age, sun exposure of skin, etc.). Some wounds heal well and barely leave scars, but some wounds have a poor prognosis and produce hypertrophic scars or keloids that affect the functional activities or aesthetics of the corresponding body parts. Notably, superficial injuries that do not reach the underlying dermis never result in keloids or hypertrophic scarring, which means that tissue repair after deep dermal injury is different from that after superficial injuries. Studies have found that when the epidermal area and the superficial part of the underlying dermis are destroyed, new epidermis will be formed from hair follicles with existing sweat and sebaceous glands (Sorrell and Caplan, 2004; Woodley, 2017). However, if the damage involves the entire thickness of the dermis, epithelialization can only be achieved by growth on the periphery of the epidermis or through the use of autografts. Therefore, we would like to further explore the boundaries that affect scar retention.

## Differences Between Fps, Frs, and F-DHJs

**FIGURE 1:**
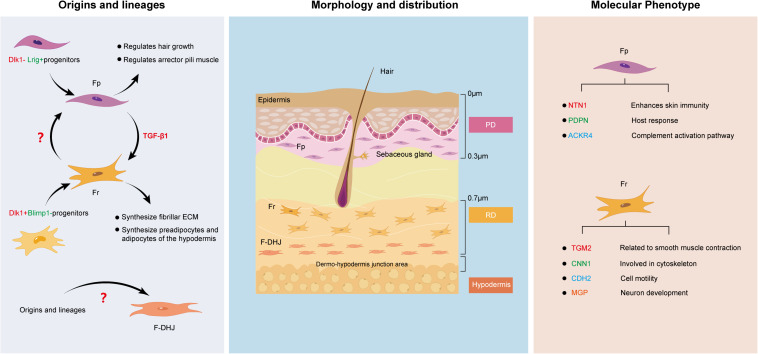
Similarities and differences between Fps, Frs, and F-DHJs: Origins and lineages, morphology and distribution, and molecular phenotype.

For a long time, fibroblasts were believed to be terminally differentiated spindle-shaped cells representing a single cell type. However, this view has since been overturned (Ravikanth et al., 2011). Various signs have shown that fibroblasts are heterogeneous in nature. One of the most obvious examples can be clearly seen in the dermis. Research consistently suggests that the dermis can be divided into two different parts, the superficial papillary layer and the deep reticular layer (Trace et al., 2016). The composition and structure of the two layers are significantly different in terms of ECM, cell density, and the structure of nerves and blood vessels, which directly contributes to study at the cellular level. Harper RA and Grove G found that fibroblasts from the papillary and reticular regions of the adult human dermis have different proliferative capacities but retain similar morphological features (Harper and Grove, 1979). The two cell types are widely known as Fps and Frs. Recently, researchers set their sights on the junction of the dermis and subcutaneous tissue, and Haydont et al. (2020) found that the fibroblasts in this area, which are named dermo-hypodermal junction fibroblasts (F-DHJs), have marked functional differences from dermal fibroblasts (Fps and Frs). This finding is perplexing to us. Currently, the boundary of the dermis is believed to be the boundary for differential healing of dermal wounds. Next, we intend to deeply analyze the similarities and differences between these three fibroblast types (Figure 1) from multiple aspects to determine the reasons for their differential tissue repair.

### Origins and Lineages

Since Fps, Frs and F-DHJs do not localize to the same region in dermal tissues, it is possible that their corresponding origins are different. Driskell et al. (2013) performed transplantation assays and lineage tracing of fibroblasts at different levels in the dermis of a mouse embryo model, and the results verified that the dermis contains two separate fibroblast lineages: Blimp1 lineage-derived fibroblasts of the papillary dermis (originating from Dlk1− Lrig+ progenitors) and Dlk1 lineage-derived fibroblasts of the reticular dermis (originating from Dlk1+ Blimp1− progenitors) (Haydont et al., 2020). The differentiation directions of the two cell lines were quite different. Additionally, lineage tracking showed that the Fp and Fr lineages do not undergo mutual transformation during skin development. This was also verified in another report. Rinkevich et al. (2015) identified the existence of two distinct embryonic fibroblast lineages that are responsible for fibrosis and non-fibrotic wound repair. The profibrotic and non-fibrotic phenotypes of Engrailed1-positive and Wnt1-positive fibroblasts were preserved after reciprocal transplantation, indicating that fibrogenic potential is an outcome of intrinsic lineage properties (Rinkevich et al., 2015).

### Morphology and Distribution

Fibroblasts are fusiform or irregular triangles, with oval nuclei in the center, cytoplasmic protrusions and radial growth (Sriram et al., 2015). Understanding their heterogeneity allows us to further divide them based on morphology and distribution. Baur et al. (1981) found that Fps exhibit a thin morphology, with bi- or tricuspid shapes and are closely arranged in the upper layers of the dermis. They are mainly distributed approximately 300–400 μm in the subepidermal dermal papillary layer, with the upper boundary highly connected with the epidermal basement membrane and the lower boundary being the vascular network of the dermal papillary layer. Frs have stellate shapes and spread morphologies and are loosely arranged with large gaps. They are located in the deep layer of the dermis and are generally found at a depth of 700 μm from the skin surface and below to avoid mixing of papillary and reticular material. In contrast, F-DHJs are more heterogeneous, with an uneven morphology, ranging from small tricuspids to larger cells that are stellate in shape with visible trabecular networks (Haydont et al., 2020). They can be harvested from the conjunctival junctions that connect the dermis to the hypodermis, where they are located.

### Molecular Phenotype

While all fibroblasts share some basic properties, primarily the ability to secrete collagen-rich ECM, no molecular markers are universally expressed by all fibroblasts, which directly leads to the dermal fibroblast subsets being historically defined by their spatial location. To date, the defining markers that allow purification of fibroblasts across skin locations and age still remain to be determined in both mouse and human skin, posing a major challenge to unbiased identification and isolation of fibroblasts. Therefore, here, we try to summarize the known surface markers of dermal fibroblasts in both mouse and human skin as comprehensively as possible, which can be seen in detail in Table 1.

**TABLE 1:** Surface markers of dermal fibroblasts from both mouse and human.

Protein name	Gene name	Fps	Frs	F-DHJs	References
Vimentin	VIM	High (H)	High (H)	High (H)	Haydont et al., 2020
Desmin	DES	Negative (H)	Negative (H)/ Negative (H)	Negative (H)	Haydont et al., 2020
Platelet derived growth factor receptor alpha	PDGFRA	Positive (M)	Positive (M)	Negative (M)	Woodley, 2017
Dipeptidyl peptidase IV (CD26)	CD26/DPP4	High (YM)/ Low (AM)	Low (YM)/ High (AM)	Low (H)	Rinkevich et al., 2015; Haydont et al., 2020
CD34	CD34	Positive (H)	Positive (H)	unknown	Haydont et al., 2020
CD36	CD36	Low (H)	High (H)	Low (H)	Korosec et al., 2019; Haydont et al., 2020
Thy-1 (CD90)	CD90/THY1	Negative (M)	Positive (M)	Positive (M)	Philippeos et al., 2018
B-lymphocyte-induced maturation protein 1	Blimp1	Positive (M)	Low (M)	Unknown	Driskell et al., 2013
Fibroblast activation protein	FAP	Positive (M)	Positive (M)	Negative (M)	Philippeos et al., 2018
Leucine rich repeats and immunoglobulin like domains	LRIG	Positive	Negative	Negative	Driskell et al., 2013; Haydont et al., 2020
Stem cells antigen 1	Sca1	Negative (M)	Negative (M)	Unknown	Driskell et al., 2013; Mascharak et al., 2020
Delta-like homolog 1	Dlk1	Negative (M)	Positive (M)	Unknown	Driskell et al., 2013
Engrailed 1	EN1	Low (M)	Positive (M)	Unknown	Mascharak et al., 2021
Aggrecan	ACAN	Low (YH)/ High (AH)	High (H)	Low (H)	Haydont et al., 2019, 2020
Collagen type XI alpha 1 chain	Col XI α1	Low (YH)/ High (AH)	High (H)	Low (H)	Haydont et al., 2019, 2020
Kruppel like factor 9	KLF9	Low (H)	Low (H)	High (H)	Haydont et al., 2020
Podoplanin	PDPN	High (H)	Low (H)	Low (H)	Korosec et al., 2019; Haydont et al., 2020
Netrin 1	NTN1	High (H)	Low (H)	Low (H)	Korosec et al., 2019; Haydont et al., 2020
Netrin 4	NTN4	Low (H)	Low (H)	High (H)	Haydont et al., 2020
α-Smooth muscle actin (α-SMA)	ACTA2	Low (H)	High (H)	Low (H)	Korosec et al., 2019; Haydont et al., 2020
Matrix gla protein	MGP	Low (H)	High (H)	Low (H)	Korosec et al., 2019; Haydont et al., 2020
Peroxisome proliferator activated receptor gamma	PPARγ	Low (H)	High (H)	Low (H)	Korosec et al., 2019; Haydont et al., 2020

Fps and Frs can be differentiated based on their unique surface marker profiles. For example, Janson et al. (2013) analyzed the specific gene expression in Fps and Frs in human skin and found that netrin-1, podoplanin and atypical chemokine receptor 4 are highly expressed in Fps, suggesting that Fps express the genes that mainly enhance skin immunity, host response and the complement activation pathway (Stunova and Vistejnova, 2018). On the other hand, high expression of transglutaminase 2, calponin1, cadherin2, and matrix gla protein in Frs indicates that Frs express genes involved in cytoskeleton dynamics and cell motility (Shinde et al., 2017; Hogervorst et al., 2018). However, there also seem to be markers, such as CD26, that change dynamically with external factors. Driskell et al. (2013) believe that CD26 marks the superior papillary dermis at the early stages of mouse development, while Rinkevich found CD26 in a large fraction of dermal fibroblasts in an adult mouse (Rinkevich et al., 2015).

## Fibroblast Heterogeneity and Tissue Repair

### Fibroblast Heterogeneity in Physiological Tissue Repair

Physiological tissue repair can also be understood as the wound healing stage, a dynamic and interactive process involving soluble mediators, blood cells, the ECM, and parenchymal cells (Rinkevich et al., 2015). This type of repair is usually summarized by three phases, hemostasis together with inflammation, granulation tissue formation and tissue remodeling, all of which occur in a temporal sequence but also overlap. Essentially, several cell types make up the wound microenvironment, and each type specializes in the performance of particular roles. When the skin is wounded, multiple cell types must coordinate at precise stages to bring about healing; thus, tissue repair is one of the most complex processes in the human body. As one of the most important components of human tissue, research on fibroblasts is ongoing. To the best of our knowledge, fibroblast heterogeneity contributes to the various functions of subpopulations in wound healing, including ECM deposition and organization, secretion of growth factors and cytokines and immunomodulation (Lian and Li, 2016; Monteran and Erez, 2019). Stunova and Vistejnova (2018) separated the intercellular communication among fibroblasts and immune cells, mast cells, keratinocytes, and endothelial cells into direct contact and autocrine or paracrine signaling. However, what role do Fps, Frs, and F-DHJs exactly play in wound healing? Are there any connections or communication between them during tissue repair? We tried to analyze these questions in the different stages of tissue repair.

Tissue injury disrupts blood vessels and causes extravasation of blood constituents. The first response of our body is constriction of the injured blood vessels and activation of platelets, which not only facilitate formation of a hemostatic plug but also lead to secretion of several mediators of wound healing, such as platelet-derived growth factor (PDGF) and transforming growth factor beta (TGF-β), that initiate the inflammatory response (Trace et al., 2016). These growth factors are important cellular mediators for the subsequent phases of wound healing. TGF-β is the central cytokine in inducing fibroblast-myofibroblast transition, and the primary task of activated myofibroblasts is to repair lost or damaged ECM (Meng et al., 2016). Janson et al. (2014) found that TGF-β1 can induce the differentiation of papillary fibroblasts to reticular fibroblasts in monolayer culture, which indicates to a certain extent that fibroblasts transformed into myofibroblasts are most likely to be Frs. PDGF is the most abundantly released factor; its paracrine effect on fibroblasts cannot be underestimated, and it is the key to the migration of fibroblasts to wounds (Feldman et al., 1993). Studies have found that Frs show greater sensitivity to PDGF than Fps, indicating that Frs are the first fibroblast type that migrates to a wound. Driskell et al. (2013) expanded on this idea, believing that with the migration of Frs, a large amount of collagen and ECM can be secreted in the early stage of wound healing.

**FIGURE 2:**
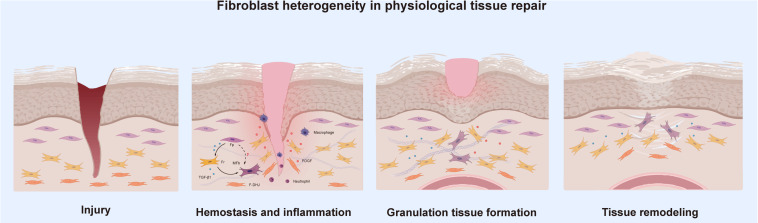
Fibroblast heterogeneity in physiological tissue repair.

As the inflammatory phase ends, the proliferative phase follows. During this phase, the healing processes synchronize, including the formation of granulation tissue, re-epithelialization, neovascularization, and immunomodulation. Granulation tissue is mainly composed of activated fibroblasts and new capillaries, with inflammatory cell infiltration (Reinke and Sorg, 2012). This tissue can not only absorb and replace various inactivated tissues to fill wounds but can also play a role in wound protection against infection. Finally, in the subsequent phase of tissue remodeling, the granulation tissue turns into scar tissue so that the wound can be repaired, marking its maturation (Poetschke and Gauglitz, 2016). Activated fibroblasts are usually myofibroblasts characterized by the expression of alpha-smooth muscle actin (α-SMA) under stimulation by profibrotic growth factors such as TGF-β1, and they are recognized as the main component of granulation tissue (Wang et al., 2008). Lineage tracing studies have revealed that initial dermal repair is attributed to lower lineage fibroblasts that express myofibroblast markers such as α-SMA, which indicates that Frs and F-DHJs participate in the “first wave” of dermal regeneration (Driskell et al., 2013; Figure 2). This also corroborates the above idea that in the hemostasis and inflammation phase, Frs migrate to the wound in the most rapid manner. Meanwhile, we also noticed an absence of Fps in the granulation tissue formation phase, and to the best of our knowledge, no studies have discussed the relationship between Fps and myofibroblasts. Given that fibroblasts are a functionally heterogeneous cell population, it is highly possible that only certain fibroblast subpopulations can differentiate into myofibroblasts during wound healing. Therefore, we speculate that Frs and F-DHJs are the main driving forces participating in repair of the dermis after being induced to myofibroblasts and that Fps may not be involved in repair of the dermis.

In most clinical settings, the closure of wounds is considered the wound healing end point, but wounds can continue to undergo remodeling or tissue maturation for several months or even years. This last stage of wound healing ultimately determines whether scarring will occur or the wound will recur. At approximately the second week of repair in genetic mouse studies, fibroblasts assume a myofibroblast phenotype characterized by ECM deposition followed by the formation of granulation tissue (Hinz, 2016a; Shook et al., 2020). As remodeling of the wound progresses, the granulation tissue matures accompanied by atrophy of blood vessels and reorganization of collagen. Collagen III lysis occurs at the same time that collagen I is synthesized, which is followed by reorganization of the ECM and the final reconstitution of granulation tissue to scar tissue (Kurkinen et al., 1980; Ehrlich, 1988). Research has found that cells from the healing dermal deep layer exhibit a phenotype resembling that of myofibroblasts in terms of expression of a-SMA and reticular markers that are associated with myofibroblasts, such as calponin 1, peroxisome proliferator-activated receptor gamma and transglutaminase 2, which directly corresponds to Frs and F-DHJs from the deep dermis and the junction (Ali-Bahar et al., 2004; Wang et al., 2008; Kulkarni et al., 2011). This finding confirms the dominant role of Frs and F-DHJs in dermal healing. In the final stage of healing, whether re-epithelialization can be achieved has an important impact on the wound healing outcome. Delayed re-epithelialization often leads to poor tissue repair. Pageon et al. (2012) used a reconstructed skin model to study the biological properties of dermal fibroblast subpopulations, and the results clearly showed that Fps have a strong ability to promote terminal keratinocyte differentiation, together with the promotion of well-structured epidermal morphogenesis. Therefore, despite being absent in repair of the dermis, in well-healed wounds, Fps participate in the final wound re-epithelization, ensuring normal repair of the tissue.

### Fibroblast Heterogeneity in Pathological Tissue Repair

**FIGURE 3:**
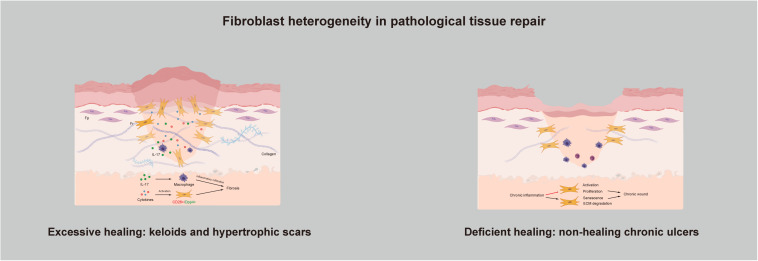
Fibroblast heterogeneity in pathological tissue repair: Excessive healing and deficient healing.

Healing is a complex and dynamic process, and scars that have no effect on either function or appearance can be regarded as the final stage of tissue repair. However, the existence of pathologic tissue repair is a concern of people worldwide. Pathologic tissue repair usually refers to two types of wound healing: Excessive healing and deficient healing (Demidova-Rice et al., 2012). As connective tissue cells, fibroblasts are responsible for collagen deposition, thus making them the main producers and organizers of the ECM and necessary for the repair of tissue injury (Hinz, 2016b). Too much collagen deposition in the wound site causes loss of normal anatomical structure and compromises function, followed by fibrosis. In contrast, deposition of an insufficient amount of collagen results in impaired wound healing (Figure 3).

#### Excessive Healing: Keloids and Hypertrophic Scars

Keloids and hypertrophic scars are characterized by excess accumulation of collagen within the wound and are typical examples of fibroproliferative disorders. They can be understood as the excessive non-functional accumulation of scar tissue. The structure of collagen fibers is poor; they have a disorderly arrangement, with no skin attachments, such as sebaceous glands and hairs. In recent years, as our understanding of the pathogenic mechanisms underlying keloid and hypertrophic scar formation has deepened, several new treatment strategies have been proposed. The key factors affecting the pathogenesis of hypertrophic scars and keloids are inflammation, fibroblasts, cytokines and ECM remodeling. They seem to be independent but are actually connected, and at the center are Frs. As previously mentioned, the reason for excessive healing resulting in skin fibrosis is that activation of Frs in the dermis leads to overproliferation and reconstruction of the ECM. If so, then what actually activates Frs? At the time when skin is injured, a large number of cytokines are secreted around the wound. They are present in very small quantities but play a significant role in directing cellular activity via chemical signals during wound healing. The migration of phagocytic neutrophils and macrophages to the wound site initiates the inflammatory phase and leads to the release of more cytokines. Researchers have found that a few cytokines function to keep the reticular dermis in the inflammatory infiltration stage in fibrotic diseases. For example, interleukin-17 was found to induce macrophage infiltration to aggravate fibrosis (Zhang et al., 2018). Dabiri et al. (2008) demonstrated that hydrogen peroxide-inducible clone 5 is an essential component of the mechanism regulating the autocrine production of TGF-β1 and the resulting pathogenic collagen contraction and ECM synthesis. Skipping treatment has also been suggested. Since chronic inflammation of the reticular dermis is the main cause of pathological scars, treatment strategies can be focused on preventing or dampening inflammation (Mizukami et al., 2020). Inflammatory infiltration in the dermis eventually activates Frs, contributing to altered ECM deposition and finally skin fibrosis. Suttho et al. (2017) designed a new reconstructed keloid model *in vitro*, which verifies our idea from another angle. The model combines fibroblasts extracted from the three major areas of a keloid (the center, periphery, and non-lesional area) in a three-dimensional biomaterial. After a series of observations and tests, they found that proliferation and collagen remodeling depend on cell origin, and they suggested that the fibroblasts from the keloid center may only be Frs. At the cellular level, through separation of scar-forming fibroblasts, researchers have found that these fibroblasts are positive for Engrailed-1 and can be separated by the surface marker CD26/DPP4, which is expressed by Frs (Rinkevich et al., 2015). Moreover, inhibition of CD26 can reduce cutaneous scarring during wound healing, which provides us with new ideas.

#### Deficient Healing: Non-healing Chronic Ulcers

In contrast with skin fibrosis caused by excessive healing, deficient healing leads to chronic wounds that do not heal for a long time; pressure ulcers, diabetic foot ulcers, and venous leg ulcers are typical examples of deficient healing (Kallis and Friedman, 2018). These injuries exhibit a disrupted repair process in which a sustained anatomical and functional result is not reached within 3 months (Diegelmann and Evans, 2004). To determine what causes this condition, researchers have examined several common components and compared them with those in normal wound healing; aging, hypoxia, ischemia-reperfusion injury, and bacterial colonization are all believed to act at the same stage of wound healing: The inflammatory phase (Bosanquet and Harding, 2014). However, clearly determining whether the observed changes, such as the abundance of neutrophils and macrophages, overproduction of reactive oxygen species (ROS), and an increase in inflammatory cytokines, are the result or the cause of the chronic wound is difficult (Zhao et al., 2016). Difficult wound healing may be caused by a lack of ECM as the organizational structure, which includes both a reduction in ECM production and excessive ECM degradation. Too much degradation can be verified by observing the changes in the inflammation phase. Studies have found that ROS overproduction causes direct damage to the ECM, and the collagenase released by neutrophils can degrade and inactivate components of the ECM (Bryan et al., 2012; Chang and Nguyen, 2021). With respect to the reduction in ECM production, the main reason for the reduction lies in the fibroblasts responsible for ECM production, which are Frs. The current view is that continued inflammation in chronic wounds inhibits the activation and proliferation of fibroblasts and induces senescence to prevent wound healing (Harding et al., 2005; Gualdi et al., 2016). Further studies have shown that a senescent cell content in ulcers exceeding 15% is associated with the chronicity of wounds and a reduced possibility of healing (Stanley and Osler, 2001). Haydont et al. (2019) conducted a genome-wide transcriptomic characterization of Fps and Frs extracted from younger and older human skin samples. Two transcripts, namely, aggrecan and collagen type XI α1, were significantly upregulated in Fps and Frs in the elderly group. Nauroy et al. (2017) initially proposed using these transcripts as Fr biomarkers, and Haydont et al. (2020) inferred that Fps may develop Fr-like characteristics with age, which can explain why chronic wounds are more likely to occur in older people.

## Dermal Fibroblast Heterogeneity Hypothesis in Wound Healing

In most cases, human skin wounds heal in a reparative way, which is called “reparative wound healing” (Woodley, 2017). This type of healing leaves scarring without the reformation of skin appendages, and one of the most representative examples is the scarring that occurs in badly burned individuals. However, there is also a type of healing that leaves no scars and has a full complement of functional skin appendages, which is called “regenerative wound healing” or “scarless wound healing” (Karppinen et al., 2019). Human fetuses have been shown to be capable of repairing skin wounds made within the first trimester of gestation without scar formation (Moore et al., 2018). Unfortunately, the mechanism of regenerative wound healing is not fully understood, and currently, achieving scarless healing is difficult. However, in-depth research on the heterogeneity of fibroblasts might provide hope for scarless healing. Frs and F-DHJs are believed to be the cells that complete the dermal remodeling stage in tissue repair, migrate to the wound site and secrete a large amount of collagen and ECM in the early stage. In addition, their expression of α-SMA and TGF-β suggests that they might be transformed into myofibroblasts. Studies demonstrating that myofibroblast apoptosis can indeed reduce scar formation suggest that approaches aimed at intervening with excess myofibroblast activation can reduce scar formation and attenuate skin fibrosis, which might be therapeutically interesting to pursue (Feng et al., 2020; Hinz and Lagares, 2020). In addition, researchers have confirmed that myofibroblasts can be reprogrammed into induced pluripotent stem cells or adipocytes (Ieda et al., 2010; Plikus et al., 2017). Thus, how can they be expanded and used in clinical applications. Would it be possible to use reprogramming to achieve mutual transformation between fibroblast subpopulations so that Fps associated with hair follicles have the ability to function in the early stages of tissue repair?

## Conclusion

Scars or skin fibrotic diseases affect everyone and are a huge burden on our country’s medical industry. Given their high degree of similarity, we noted the bond that links these conditions together: Fibroblasts. The study of fibroblasts helps us to more deeply understand the heterogeneity of fibroblasts and their subpopulations, including Fp, Fr, and F-DHJ subpopulations. Each subpopulation has its own unique physiological characteristics and plays a corresponding role in the skin microenvironment in either an autocrine or paracrine manner.

The early involvement of Frs in wound healing and their similarity to myofibroblasts give us reason to believe that they play a key role in reparative wound healing. In addition, the lack of Fps associated with hair follicles in the early stage of wound healing has also become a new idea. Multiple related studies must be carried out, and we believe that there are very broad application prospects in tissue repair and functional reconstruction.

## Author Contributions

F-LY designed the study, reviewed, and revised the manuscript. M-LZ, Y-YT, J-JW, and X-YT drafted the manuscript. Z-LS, S-YL, YJ, Z-HC, and K-WZ critically reviewed the studies. M-LZ, XL, J-XY, and R-SX performed the systematic review. All authors contributed to the article and approved the submitted version.

## Conflict of Interest

The authors declare that the research was conducted in the absence of any commercial or financial relationships that could be construed as a potential conflict of interest.

## Abbreviations

Fps: papillary fibroblasts
Frs: reticular fibroblasts
F-DHJs: dermal-subcutaneous junction fibroblasts
ECM: extracellular matrix
Dlk1: delta-like homolog 1
Blimp1: b-lymphocyte -induced maturation protein-1
PDGF: platelet-derived growth factor
TGF-β: transforming growth factor beta
α-SMA: alpha-smooth muscle actin
ROS: reactive oxygen species.
